# Comparison of 7-site skinfold measurement and dual-energy X-ray absorptiometry for estimating body fat percentage and regional adiposity in Taiwanese diabetic patients

**DOI:** 10.1371/journal.pone.0236323

**Published:** 2020-07-24

**Authors:** Feng-Chih Kuo, Chieh-Hua Lu, Li-Wei Wu, Tung-Wei Kao, Sheng-Chiang Su, Jhih-Syuan Liu, Kuan-Chan Chen, Chia-Hao Chang, Chih-Chun Kuo, Chien-Hsing Lee, Chang-Hsun Hsieh

**Affiliations:** 1 Division of Endocrinology and Metabolism, Department of Internal Medicine, Tri-Service General Hospital, National Defense Medical Centre, Taipei, Taiwan, ROC; 2 Division of Family Medicine, Department of Family and Community Medicine, Tri-Service General Hospital, and School of Medicine, National Defense Medical Center, Taipei, Taiwan, ROC; 3 Division of Geriatric Medicine, Department of Family and Community Medicine, Tri-Service General Hospital, and School of Medicine, National Defense Medical Center, Taipei, Taiwan, ROC; 4 Graduate Institute of Medical Sciences, National Defense Medical Center, Taipei, Taiwan, ROC; San Raffaele Roma Open University, ITALY

## Abstract

Obesity and regional adiposity are important risk factors for cardiometabolic disorders. The aim of this study is to compare 7-site skinfold (SF) measurement to dual-energy x-ray absorptiometry (DXA) as the reference method for estimating body fat percentage (BF%) and regional adiposity in diabetic outpatients. A total of 59 diabetic patients (36 females and 23 males) aged 28.5–78 years (median 67.7 years) with BMI 18.8–40.6 kg/m^2^ (median: 25.5 kg/m^2^) were enrolled. 7-site skinfold measurement and DXA were performed at the same visit day and biochemistry data were collected. Our results demonstrate the BF% calculated via Jackson & Pollock 7-site skinfold equation presents a strong correlation (*r* = 0.672, *p* < 0.001 in females; *r* = 0.885, *p* < 0.001 in males) with that measured by DXA, but the means of BF% between these two methods are significantly different in both sexes (paired *t*-test, *p* < 0.001). The Bland-Altman analysis showed the mean differences (DXA-SF) of BF% were positive for female (8.74%) and male (7.22%), suggesting Jackson & Pollock 7-site skinfold equation tends to underestimate the BF%. Besides, regional SF thicknesses of 7-site skinfold measurement were significantly correlated with the matched regional adiposity quantified by DXA. Furthermore, truncal and android SF thicknesses were notably positively correlated with several cardiometabolic risk factors in gender-specific manner. Our data indicate the 7-site skinfold measurement is not an interchangeable method for precisely measuring BF%, but might be practical for evaluating the cardiometabolic risks in Taiwanese diabetic outpatients.

## 1. Introduction

Obesity is a global healthy issue to an extent that almost a third of the world’s population is nowadays classified as overweight or obese [1]. In Taiwan, according to three consecutive waves of Nutrition and Healthy survey (1993–1996, 2005–2008, and 2013–2014), the prevalence of obesity (BMI ≥27 kg/m^2^) increased sharply from 11.8%, 17.9%, to 22.0% and morbid obesity (BMI ≥35 kg/m^2^) elevated from 0.4%, 0.6%, to 1.4% [2]. Besides general adiposity, regional adiposity: such as central obesity is well proven as a harmful phenotypic feature associated with increased risks for diabetes, cardiovascular diseases and cancer [3]. Notably, a recent large cohort study in Korean has assessed the differential impacts of general and regional adiposity on major adverse cardiac events (MACE) and found abdominal adiposity alone was associated with an increased risk of MACE, whereas general obesity without abdominal adiposity did not increase the risk [4]. The underlying pathogenic mechanisms are closely related to subcutaneous adipose tissue dysfunction with subsequent overspill, ectopic deposition of visceral adipose depots, which release pro-inflammatory adipocytokines causing chronic inflammation and insulin resistance [5–7]. In diabetic patients, the prevalence of overweight and obesity is even higher [8] and injection therapy with insulin will almost inevitably exacerbate body weight gain. With the increase of body weight, the diabetic patients further encounter augmented risks for diabetic morbidity and mortality [9]. Therefore, beyond the conventional measurements of body mass index or waist-to-hip ratio, there is still an urgent need to have a practical tool for evaluating the body fat percentage (BF%) and regional adiposity in diabetic patients under regular outpatient follow-up.

Dual-Energy X-ray Absorptiometry (DXA) scanning was considered as a reference method for measuring different body composition such as total lean mass, bone mineral content, total fat mass and BF% [10, 11]. DXA could also precisely quantify regional fat mass and lean mass. Regarding the regional lean mass quantification, appendicular skeletal muscle mass divided to BMI or square of height has been applied as one of the diagnostic criteria for sarcopenia [12–14]. Additionally, DXA could delicately analyze human body fat distribution. The gynoid fat mass adjusted for total fat mass has been showed negatively associated with several cardiovascular risk factors, such as hyperlipidemia and insulin resistance [15]. These findings are in line with previous large epidemiologic survey using hip circumference as the surrogate of lower body adiposity, which demonstrated hip circumference is independently associated with lower risk for myocardial infarction [16]. Hence, the gluteofemoral fat was considered as a protective metabolic sink, which stably stores triglycerides to alleviate the ectopic fat deposition and lower the cardiometabolic risks [17]. In contrast, increase of android fat and visceral fat (central adiposity) is independently correlated with higher risk for cardiovascular disease [5, 6, 15, 18]. However, the gold standard method: DXA for quantifying BF% and regional fat mass is not generally accessible in the outpatient clinic since the machine is expensive, space occupied, experienced operator required and radiation exposure involved.

Skinfold thickness measurement has been applied to evaluate fatness for over 50 years and is cheap, handy in the outpatient clinic [19]. It was performed via pinching the subcutaneous skinfold in different sites and measuring the thickness with specialized calipers. Studies have revealed it is appropriate for comparing regional adiposity [20–22]. Several equations have also been developed for calculating BF% using the raw data of skinfold thickness under the assumptions that majority of adipose tissues were located in the subcutaneous area and the body fat is equally distributed over the body [23–26]. However, this technique required practice for performing the standardized measurement and was disputed by high variations between observers. Currently, studies assess the clinical significance of these skinfold parameters are rare [27, 28].

Since obesity, particular central adiposity, will significantly impede the glucose control and increase cardiovascular risks in diabetic patients, assessment of BF% and body fat distribution is crucial. Therefore, we aim to investigate the application of 7-site skinfold measurement and compare it with DXA for estimating BF% and fat distribution in diabetic patients under regular outpatient follow-up.

## 2. Materials and methods

In total, 59 adults (23 males and 36 females) who receive regular follow-up in outpatient department of Endocrinology and Metabolism, Tri-Service General Hospital were recruited. The criteria for inclusion into this trial were as follows: age 25–80 years with type 2 diabetes mellitus under stable control with either oral hypoglycemic agents or injection therapy of insulin or glucagon-like peptide 1 receptor agonist. The exclusion criteria were pregnancy, current acute illness of cerebrovascular accident, myocardial infarction, heart failure, renal failure, hepatic failure or psychiatric diseases. All participants signed written informed consent before participating in this study and agreed to the use of relevant personal information on a confidential basis. The institutional review boards of Tri-Service General Hospital (TSGH) approved this study. (TSGHIRB number: 2-108-05-052)

### Anthropometric measurements

Body weight was measured to the nearest 0.1 kg; body height, waist circumference and hip circumference were measured to the nearest 0.1 cm; waist-to-hip circumference ratio and body mass index (BMI) were calculated. Body weight and standing height were detected using a standard scale and a wall-mounted stadiometer, respectively as barefoot with the patients wearing light indoor clothing. Waist circumference was measured at the midway horizontal plane between the inferior margin of the last rib and the crest of the ilium. Hip circumference was measured at its widest point. BMI was calculated as weight in kilograms divided by the square of height in meters. Blood pressure was measured from the right arm in a sitting position after resting for 5 minutes. One minute later, the blood pressure was measured again and the average value was used in the analysis.

### 7-site skinfold thickness measurement

The standard protocols of skinfold measurement are according to the recommendations published by the Committee on Nutritional Anthropometry of the Food and Nutrition Board of the National Research Council [29]. The skinfold thicknesses were measured to the nearest 0.1 mm using Lange skinfold caliper. Two readings with the difference less than 2 mm were recorded and the average values were used for analysis. All the skinfold measurements in these diabetic patients were performed by a single experienced technician to avoid the variation between observers. Total 7-different sites of the right side body were measured in each individual, which include tricep, subscapular, chest, midaxillary, suprailiac, abdominal and thigh skinfold thicknesses and Jackson & Pollock 7-site skinfold equation was used for calculating the BF% [24, 25]. The sum of suprailiac and abdominal skinfold thickness was further referred as the android skinfold thickness. The tricep-to-android skinfold ratio and thigh-to-android skinfold ratio were applied as the surrogates for peripheral fat distribution.

### Dual-energy X-ray absorptiometry

Dual-energy X-ray absorptiometry (DXA) was used as a standard reference for measuring whole and regional body composition, including fat mass, lean mass, and bone mineral content. Participants were dressed in cotton robes without metal attachments, lying in a supine position in the center of the scanning field with their palms facing downwards, arms positioning away from their body, and feet, face, chin all maintaining in the neutral position. The scan took around 5–10 mins to complete, and the dose of radiation per individual was less than 0.01mGy (1.0 mrad). The composition of different body regions including arms, legs, android, gynoid and trunk was measured in grams by the DXA software (enCORE V13.60.033). The type of the DXA machine is Lunar Prodigy Advance enCORE 2011.

### Biochemical variables measurement

Venus blood samples were drawn following an 8-hour fast. Levels of glucose, lipids, liver and renal function were then measured. Serum levels of total cholesterol, triglycerides, low-density lipoprotein (LDL) cholesterol, alanine aminotransferase (ALT) and creatinine were measured using a Beckman Synchron LX20 analyzer (LX20; Beckman Coulter, Brea, CA, USA). Plasma glucose concentrations were determined using the glucose oxidase method on a Beckman Glucose Analyzer II (Beckman Instruments, Fullerton, CA, USA). Before measuring the biochemical variables, calibration and quality control with standard solutions will be performed ahead. Then, the measurement will be repeated provided abnormal data were observed. Biochemistry data were collected from recent 3-months medical records with average 34.7 ± 27.5 days (mean ± standard deviation) away from the date of DXA, anthropometric and skinfold measurements.

### Statistical analysis

Continuous variables were analyzed using the Mann-Whiney U-test and presented as median values with quartiles. Chi-square test was applied for assessing the categorical variables and presented as percentages. Statistical significance was defined as p value less than 0.05, but was adjusted with Bonferroni correction as needed. Correlations of estimated BF% and body fat distribution between 7-site skinfold measurement and DXA were assessed using Spearman rank-order correlations. Agreement of BF% between two methods were evaluated with Bland-Altman analysis [30] and presented with mean, standard deviation (SD) and 95% limits of agreement (LOA). Mean of between-methods differences in BF% was examined using paired sample t-test. Concordance of BF% between 7-stie skinfold measurement and the standard DXA was further analyzed with Lin’s concordance correlation coefficients [31]. Correlations between skinfold-caliper measured regional adiposity and biochemistry data were also determined using Spearman rank-order correlations in gender-specific manner. All statistical analyses were performed using SPSS software version 22 (IBM, Chicago, Illinois, USA).

## 3. Results

Gender specific comparisons of the basic anthropometric variables, biochemical characteristics and status of diabetic treatment were shown in Table 1. Serum creatinine level is significantly higher in males than that in females. There are no significant difference between genders in age, BMI, waist-to-hip ratio and blood pressure. The median history of diabetes is 10 years in both male and female group. The status of diabetes was well controlled in both sexes without significant difference in the levels of fasting glucose and HbA1c. The prescriptions of statin, oral hypoglycemic and injectable anti-diabetic agents are all similar in both groups. Notably, anti-diabetic agents that potentially could influence body weight or fat distribution, such as thiazolidinedione (TZD), sodium-glucose co-transporter 2 inhibitor (SGLT2i), insulin and glucagon-like peptide 1 receptor agonist (GLP1RA) were prescribed in both groups without significant difference.

**Table 1:** Basic anthropometric, biochemical characteristics and status of diabetic treatment in the study population.

	Females	Males	*p* value
	(n = 36)	(n = 23)	
Age (years)	67.6 [63.9; 71]	67.7 [60.4; 71.4]	0.816
BMI (kg/m^2^)	25.0 [21.9; 28.2]	26.1 [23.5; 31.4]	0.164
Waist-to-hip ratio	0.94 [0.91; 1.00]	0.96 [0.92; 1.03]	0.181
Systolic BP (mmHg)	132 [120; 145]	136 [125; 146]	0.491
Diastolic BP (mmHg)	74 [66; 82]	79 [72; 83]	0.168
Fasting glucose (mmol/L)	7.10 [5.77; 8.27]	6.72 [5.88; 8.16]	0.988
HbA1c (%)	7.2 [6.6; 7.8]	7.1 [6.6; 8.0]	0.744
Total cholesterol (mmol/L)	4.12 [3.47; 4.30]	3.65 [3.37; 4.17]	0.181
LDL cholesterol (mmol/L)	2.31 [1.79; 2.72]	1.97 [1.76; 2.54]	0.283
Triglyceride (mmol/L)	3.06 [1.97; 3.68]	2.38 [1.97; 2.98]	0.244
Creatinine (μmol/L)	61.9 [53.0; 70.7]	88.4 [70.7; 106.1]	<0.001**
ALT (U/L)	17 [15; 25]	19 [13; 27]	0.892
Statin (%)	80.6%	60.9%	0.097
Metformin (%)	72.2%	82.6%	0.360
AGI (%)	11.1%	17.4%	0.699
SU or glinide (%)	52.8%	52.2%	0.964
TZD (%)	8.3%	8.7%	1.000
SGLT2i (%)	25%	21.7%	0.774
DPP4i (%)	30.6%	34.8%	0.735
GLP1RA (%)	13.9%	17.4%	0.725
Insulin (%)	38.9%	39.1%	0.985
History of diabetes (years)	10 [5.13; 15]	10 [4; 19]	0.882

Continuous variables were analyzed using the Mann-Whitney U-test and are presented as median values and [quartiles]; Categorical variables were analyzed using the Chi-square test and are presented as percentages. Abbreviations: BMI, body mass index; BP, blood pressure; HbA1c, glycated hemoglobin; LDL, low density lipoprotein; ALT, alanine aminotransferase; AGI, alpha glucosidase inhibitor; SU, sulfonylurea; TZD, thiazolidinedione; SGLT2i, sodium-glucose co-transporter 2 inhibitor; DPP4i, dipeptidyl peptidase 4 inhibitor; GLP1RA, glucagon-like peptide 1 receptor agonist.

**p<0.001

In Table 2, 7-site skinfold measurement and DXA associated variables were listed and compared between genders. Males have significantly higher chest skinfold thickness, higher total lean mass and higher total bone mineral content than those in females. In contrast, females present significantly higher peripheral-to-android fat distribution and total body fat%, which were consistently demonstrated by either 7-site skinfold measurement or DXA scanning. Regarding the regional fatness in extremities and android region, there are no significant differences between genders in both methods. Notably, females tend to have higher (non-significant) fat mass and skinfold thickness in extremities than those in males, which again present compatible findings between two methods.

**Table 2:** Adiposity and body composition evaluated by 7-site skinfold (SF) measurement and dual-energy X-ray absorptiometry (DXA) scan.

	Females	Males	*p* value
	(n = 36)	(n = 23)	
**Skinfold measurement**			
Chest SF (mm)	4.5 [3.8; 6.1]	5.8 [4.8; 8.8]	0.013*
Abdominal SF (mm)	24.0 [18.4; 28.0]	26.0 [20.0; 35.0]	0.186
Thigh SF (mm)	17.9 [13.6; 24.5]	13.5 [9.25; 27.0]	0.132
Tricep SF (mm)	21.0 [17.5; 23.6]	14.3 [11.8; 28.5]	0.101
Subscapular SF (mm)	20.8 [15.3; 22.9]	20.3 [17.2; 23.6]	0.736
Suprailiac SF (mm)	19.3 [16.1; 23.4]	16.0 [14.0; 24.0]	0.410
Midaxillary SF (mm)	18.3 [14.8; 24.0]	16.8 [12.5; 26.5]	0.756
Android SF (A+Sup) (mm)	42.1[33.4; 52.8]	42.0 [34.3; 59.0]	0.680
Tricep-to-android SF ratio	0.47 [0.40; 0.58]	0.38 [0.32; 0.49]	0.009*
Thigh-to-android SF ratio	0.44 [0.32; 0.59]	0.33 [0.29; 0.40]	0.031*
Body fat-SF (%)	26.9 [23.3; 30.2]	21.5 [18.3; 27.3]	0.003*
**DXA scan**			
Total lean mass (kg)	36.4 [32.4; 39.3]	50.7 [45.7; 54.4]	<0.001**
Total BMC (kg)	1.93 [1.66; 2.23]	2.78 [2.44; 2.92]	<0.001**
Total fat mass (kg)	20.8 [15.1; 25.1]	21.0 [13.4; 31.8]	0.913
Arms fat mass (kg)	2.21 [1.56; 2.85]	1.49 [1.22; 2.51]	0.082
Legs fat mass (kg)	4.84 [3.61; 6.48]	3.99 [3.17; 6.63]	0.382
Android fat mass (kg)	2.20 [1.49; 2.86]	2.52 [1.51; 3.86]	0.277
Arms-to-android fat ratio	0.99 [0.85; 1.20]	0.69 [0.54; 0.80]	<0.001**
Legs-to-android fat ratio	2.35 [1.99; 3.06]	1.90 [1.69; 2.17]	0.006*
Body fat-DXA (%)	36.5 [30.3; 40.9]	28.5 [20.7; 39.0]	0.009*

Continuous variables were analyzed using the Mann-Whitney U-test and are presented as median values and [quartiles]. Abbreviation: SF, skinfold; DXA, dual-energy X-ray absorptiometry; A, abdominal; Sup, suprailiac; BMC, bone mineral content. Android skinfold was referred as the sum of abdominal and suprailiac skinfold thickness. Body fat-SF (%) was calculated by Jackson & Pollock 7-site skinfold equation.

*p<0.05;

**p<0.001.

**Fig 1:**
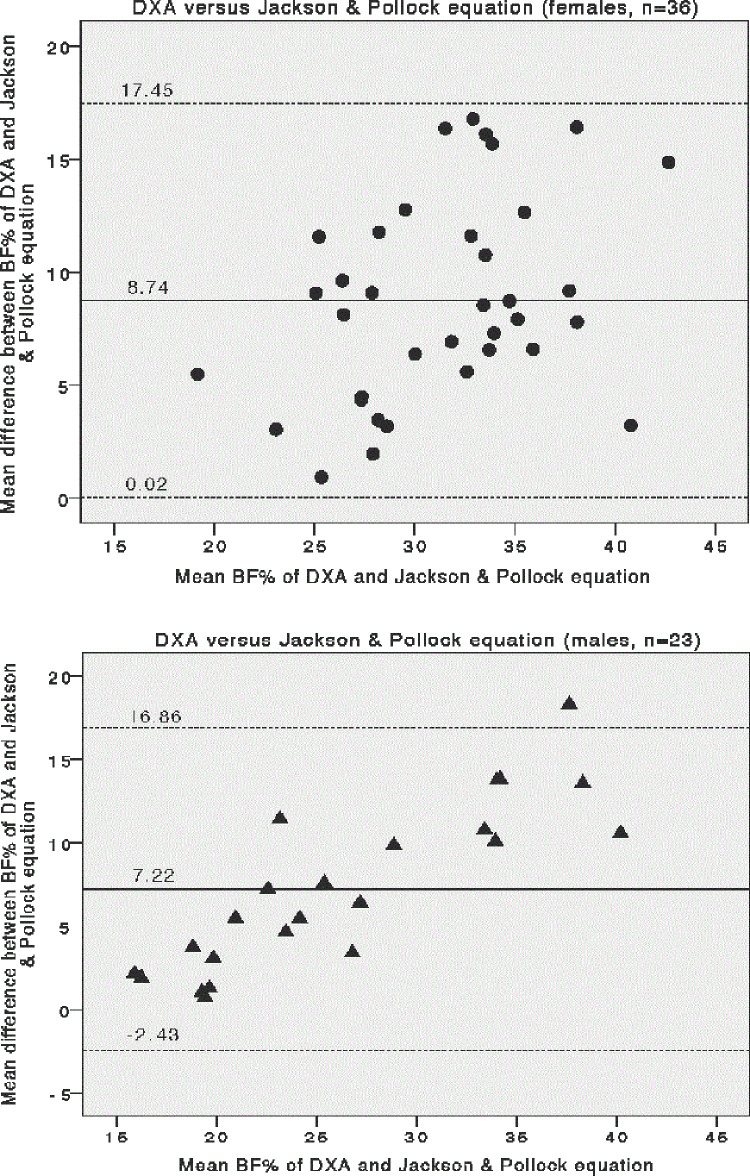
Comparison of BF% measured by DXA and Jackson & Pollock 7-site skinfold equation and displayed as Bland–Altman plots in females (a) (black circle) and males (b) (black triangle). The central line represents the mean bias between measurements. Dotted lines represent upper and lower 95% limits of agreement. Y-axis represents difference of BF% measured by DXA minus BF% measured from skinfolds. Abbreviations: BF%, body fat percentage; DXA, dual energy X-ray absorptiometry.

In order to assess the correlation and agreement between two methods for measuring body fat% (DXA and Jackson & Pollock 7-site skinfold equation), Spearman correlation, Lin’s concordance correlation coefficient and Bland-Altman analysis were performed as shown in Table 3 and Fig 1. The BF% calculated from Jackson & Pollock 7-site skinfold equation shows strong correlations with the BF% measured by the standard DXA with greater in males (*r* = 0.885, *p* < 0.001) than females (*r* = 0.672, *p* < 0.001). However, the absolute BF% values estimated by two methods only present fair concordance in female (*ρc* = 0.307, 95%CI = 0.162–0.439) and moderate concordance in males (*ρc* = 0.561, 95%CI = 0.380–0.700). The means of BF% measured by these two methods are significantly different in both sexes (paired *t*-test, *p* < 0.001). The Bland-Altman analysis revealed the mean differences (DXA-SF) of BF% are positive for female (8.74%) and male (7.22%) with respective 95%LOA: 0.02% to 17.45% and -2.43% to 16.86%, indicating Jackson & Pollock 7-site skinfold equation tends to underestimate the BF%.

**Table 3:** Spearman correlation, CCC, and Bland–Altman analysis in body fat% measured by two methods: DXA and Jackson & Pollock 7-site skinfold (SF) equation.

	Total (n = 59)	Females (n = 36)	Males (n = 23)
Spearman correlation (*r*)	0.837**	0.672**	0.885**
CCC (ρc (95%CI))	0.456 (0.338–0.560)	0.307 (0.162–0.439)	0.561 (0.380–0.700)
Bland-Altman analysis			
Bias (DXA-SF)	8.14**	8.74**	7.22**
SD	4.66	4.45	4.92
95% LOA	-0.98 to 17.27	0.02 to 17.45	-2.43 to 16.86

DXA, dual-energy X-ray absorptiometry; SF, skinfold; *r*, correlation coefficient; CCC, Lin’s concordance correlation coefficient; SD, standard deviation; LOA, limits of agreement. Bias was referred as mean of between-methods differences in body fat% and was examined using paired sample *t*-test.

**p<0.001.

Comparison between 7-site skinfold measurement and DXA in assessment of regional adiposity was executed in gender-specific manner as shown in Table 4. In both sexes, there are significantly high correlations between two methods regarding assessment of fatness in arms, legs and android region. In females, the strongest correlation (*r* = 0.76) was observed in legs region, whereas males have the strongest correlation (*r* = 0.89) in android region. When assessing the peripheral-to-android fat distribution, there is significantly moderate correlation between two methods, solely in the legs-to-android fat ratio of females (*r* = 0.49).

**Table 4:** Gender-specific correlations in regional adiposity assessed by two methods: DXA and 7-site skinfold measurement.

	DXA: Arms fat	Legs fat	Android fat	Arms/android fat ratio	Legs/android fat ratio
Tricep SF	(0.57*; 0.68*)				
Thigh SF		(0.76*; 0.84*)			
Android SF			(0.72*; 0.89*)		
Tricep/android SF ratio				(0.42; 0.02)	
Thigh/android SF ratio					(0.49*; 0.42)

Data were analyzed with Spearman correlation and presented with gender-specific correlation coefficients (female *r*; male *r*). Abbreviation: DXA, dual energy X-ray absorptiometry; SF, skinfold; BMI, body mass index. Android skinfold was referred as the sum of abdominal and suprailiac skinfold thickness.

*p value less than 0.01 was deemed significant, after the Bonferroni adjustment

Correlations between different skinfold variables with age, BMI, blood pressure and biochemistry data were analyzed in females and males, separately as shown in Table 5. All skinfold variables are significantly positively correlated with BMI, except chest skinfold thickness and the peripheral (tricep or thigh) to android skinfold ratios. In females, the peripheral (tricep or thigh) to android skinfold ratios are negatively correlated with age. In both genders, the skinfolds in trunk (except chest skinfold) and android region are positively correlated with cardiometabolic disorders related parameters including blood pressure, glucose levels, lipid profiles and liver function. Whereas, the positive correlations with HbA1c were only observed in females and males present strong correlations with systolic blood pressure, diastolic blood pressure and ALT level. Intriguingly, the peripheral (tricep or thigh) to android skinfold ratios are free from any correlation with blood pressure and these biochemistry data. Also, the female thigh skinfold thickness is not correlated with blood pressure and any of these biochemistry data.

**Table 5:** Gender-specific correlations between skinfold variables, age, BMI and several cardiometabolic risk factors.

**Female**	Tri-SF	Sub-SF	Che-SF	Mid-SF	Sup-SF	Abd-SF	Thi-SF	And-SF	Tri/And	Thi/And
Age	0.27	-0.17	-0.17	-0.17	0.15	0.08	-0.20	0.10	-0.43**	-0.36*
BMI	0.55**	0.70**	0.17	0.69**	0.71**	0.60**	0.50**	0.68**	-0.16	0.15
SBP	-0.06	0.23	0.002	0.18	0.15	0.08	-0.02	0.14	-0.10	-0.06
DBP	0.14	0.36*	0.28	0.35*	0.29	0.22	0.28	0.31	-0.04	0.15
FG	0.17	0.31	-0.01	0.17	0.20	0.17	-0.18	0.23	-0.17	-0.28
HbA1c	0.23	0.42*	0.01	0.43**	0.55**	0.37*	0.10	0.49**	-0.27	-0.16
TC	0.16	0.12	0.24	0.09	0.13	0.17	0.02	0.18	0.03	-0.08
LDL-C	0.27	0.40*	0.25	0.36*	0.28	0.27	-0.09	0.29	0.01	-0.28
TG	0.24	0.22	0.18	0.18	0.31	0.33	0.25	0.36*	-0.03	0.15
Cr	-0.03	-0.26	-0.01	-0.30	-0.05	-0.01	0.12	-0.05	-0.01	0.14
ALT	0.40*	0.53**	0.29	0.43**	0.24	0.18	0.27	0.24	0.25	0.22
**Male**	Tri-SF	Sub-SF	Che-SF	Mid-SF	Sup-SF	Abd-SF	Thi-SF	And-SF	Tri/And	Thi/And
Age	-0.29	-0.12	-0.24	-0.22	-0.27	-0.32	-0.06	-0.31	0.08	0.03
BMI	0.54**	0.91**	0.18	0.83**	0.71**	0.81**	0.62**	0.79**	-0.13	0.11
SBP	0.68**	0.78**	0.10	0.81**	0.74**	0.64**	0.57**	0.69**	0.23	0.16
DBP	0.50*	0.42*	0.09	0.52*	0.59**	0.58**	0.32	0.57**	-0.03	0.08
FG	0.42*	0.13	-0.01	0.11	0.04	0.07	-0.08	0.07	0.35	-0.20
HbA1c	-0.01	-0.21	0.18	-0.23	-0.18	-0.23	0.06	-0.19	0.06	0.27
TC	-0.09	-0.05	0.05	0.08	0.07	0.06	0.04	0.10	-0.15	-0.02
LDL-C	-0.18	-0.02	-0.12	0.14	0.14	0.13	0.01	0.10	-0.32	-0.20
TG	0.21	0.38	-0.04	0.37	0.38	0.48*	0.45*	0.46*	-0.34	0.17
Cr	0.18	0.36	-0.04	0.24	0.23	0.23	0.14	0.24	-0.04	-0.09
ALT	0.37	0.48*	0.14	0.55**	0.62**	0.75**	0.42*	0.72**	-0.41	-0.01

Data were analyzed with Spearman correlation and presented with gender-specific correlation coefficients (*r*). Abbreviation: SF, skinfold; Tri, tricep; Sub, subscapular; Che, chest; Mid, midaxillary; Sup, suprailiac; Abd, abdominal; Thi, thigh; And, android; Tri/And, tricep-to-android skinfold ratio; Thi/And, thigh-to-android skinfold ratio; BMI, body mass index; SBP, systolic blood pressure; DBP, diastolic blood pressure; FG, fasting glucose; HbA1c, glycated hemoglobin; TC, total cholesterol; LDL-C, low density lipoprotein cholesterol; TG, triglyceride; Cr, creatinine; ALT, alanine aminotransferase. Android skinfold was referred as the sum of abdominal and suprailiac skinfold thickness.

*p<0.05;

**p<0.01.

## 4. Discussion

Our results indicate that 7-site skinfold measurement might be a practical method for assessing the body fat percentage, regional adiposity and cardiometabolic risks in Taiwanese diabetic outpatients. In Table 3, the BF% calculated via Jackson & Pollock 7-site skinfold equation showed a strong correlation with that measured by the standard method: DXA. However, the absolute values of BF% measured by these two methods are not concordant. Jackson & Pollock 7-site skinfold equation usually underestimates the BF%. Previous studies comparing the BF% estimated by other skinfold equations and DXA also report similar findings that the skinfold method tends to undervalue the actual BF% [32, 33], whereas it might still depend on which skinfold equation was applied [34]. Besides, the referenced database for developing the Jackson & Pollock skinfold equation is based on the Caucasian population [24, 25]. Therefore, understanding the mean differences of BF% between the skinfold method and standard DXA will provide useful information for adjusting the BF% calculated via Jackson & Pollock 7-site skinfold equation in Taiwanese diabetic patients. While, to establish the adjusted skinfold equation fitting in our population will require further large database and could not be achieved in this study.

The potential utilization of skinfold measurement for evaluating the regional adiposity or fat distribution has been reported in some studies. Ketel et al. [20] found combining skinfold measurements with waist is superior to the waist-to-hip ratio for determining the body fat distribution in Caucasian Dutch adults. Also, Surendar et al. [21] observed the skinfold measurements of trunk fat were higher in the South Indian people with parental history of diabetes than those with no parental history. Moreover, measuring the skinfold thickness over multiple body sites in 40–60 year-old Bulgarian women found the diabetic females presented primarily upper torso fat distribution and less so in the limbs comparing to the healthy females [22]. In our study, we assess the body fat distribution via calculating the ratio between peripheral skinfold (tricep or thigh skinfold) and android skinfold (sum of abdominal and suprailiac skinfolds). Intriguingly, both the tricep or thigh-to-android skinfold ratio and DXA defined arms or legs-to-android fat ratio are higher in women than men, supporting the usefulness of skinfold measurement in assessing fat distribution while these patients have had around 10-years history of type 2 diabetes (Table 2). Furthermore, the gender-specific correlations between the regional skinfold thickness and DXA defined regional fat mass were directly evaluated in the arms, legs and android regions. The calculated peripheral-to-android ratios were also specifically assessed (Table 4). Overall, the gender-specific correlations of these two methods in regional fatness are moderate to very strong (Spearman correlation coefficients between 0.57 to 0.89). Notably, in males, the strongest correlation was observed in the android fatness (r = 0.89) and, in females, the strongest correlation was showed in the legs fatness (r = 0.76), which is in line with the gender-specific differences in apple shape or pear shape body figures [35]. It seems the predominant body phenotype is easier to be identified when using the caliper to measure skinfold thickness. Indeed, regarding the peripheral-to-android fat distribution, only females showed a significant correlation when comparing DXA defined legs-to-android fat ratio to the thigh-to-android skinfold ratio measured by 7-site skinfold method (Table 4). These results point out the 7-site skinfold measurement could be applied for examining the regional adiposity and fat distribution in the diabetic outpatients under regular follow-up.

Currently, only few studies investigate the associations of regional body fat distribution or skinfold thickness with the cardiometabolic risk factors. A previous study in 43,595 women via examining the relation of DM with six girths found upper body (waist, bust, and neck girths) was positively related to the prevalence of DM, whereas DM was inversely related to the lower body (hip and ankle girths) [36]. Lee et al. [37] analyzed 2306 participants (mean aged 60 years, 54.4% women) who underwent computed tomography from the Framingham Heart Study between 2008–2011 and observed higher upper body subcutaneous fat is associated with adverse cardiometabolic risk factors, including increase in systolic blood pressure, fasting plasma glucose and triglycerides with decrease of high-density lipoprotein cholesterol. Similar analysis has been performed in Korean National Health and Nutrition Examination Surveys (2008–2010) using dual-energy X-ray absorptiometry in around 7000 individuals (54.6% women) aged 50 years or older, which revealed increase of truncal and arm fat mass was related to higher odds ratios for diabetes mellitus (DM), while higher leg fat mass was associated with a lower risk of DM [38]. Consistently, Pinnick et al. [15] evaluate DXA quantified regional fat mass in a healthy population-based cohort (n = 3,399, aged 29–54 years) and gynoid fat mass after adjustment for total fat mass is inversely associated with insulin resistance, dyslipidemia and hypertension in both genders. Regarding to assess the associations of skinfold thickness with the cardiometabolic risk factors, Hariri et al. [28] performed upper body (tricep, bicep, subscapular and suprailiac) skinfold measurement in white males and the strongest correlations were seen with the serum triglyceride levels (positive correlation) and indices of insulin sensitivity (negative correlation). Similar observation was also reported by Addo et al. [27] that, in US adolescents, tricep and subscapular skinfold thickness were comparable with DXA whole-body fat in predicting serum triglyceride levels. Moreover, a prospective study conducted in 988 adult Peruvian found sub-scapular skinfold thickness was strongly associated with the development of type 2 DM and hypertension [39]. However, none of these studies concurrently evaluate both upper and lower body skinfold thickness in diabetic patients under regular outpatient follow-up.

In our study, we performed 7-site skinfold measurements in diabetic outpatients and observe similar findings (Table 5) that upper body fatness, particularly truncal and android skinfold thickness were positively correlated with HbA1c levels in women and positively correlated with systolic blood pressure, diastolic blood pressure, ALT levels in men. These gender-differential correlations are intriguing, but still required further investigation to ascertain these observations. Previous research has showed, for a given body mass index, females with phenotypic pear-shaped adiposity usually could maintain better insulin sensitivity than males [40]. Therefore, it seems reasonable when elder women had dysregulated upper body adiposity might particularly suffer from glucose dysregulation as observed in our data. Also, males with central adiposity commonly will be vulnerable to the visceral fat accumulation [41]. Since the visceral fat deposition will increase hepatic fatty infiltration and induce systemic inflammation, it won’t be surprised that males with increased truncal or android adiposity were positively correlated with elevated blood pressure and liver function. Whereas, the lower body fatness presented by thigh skinfold thickness in women is free of correlation with any blood pressure and these biochemistry data. Notably, the peripheral (tricep or thigh) to android skinfold ratio is also free of correlation with these cardiometabolic risk factors in both genders. However, this cross-sectional observation is just an exploratory assessment and will definitely require a larger population with a prospective cohort study to validate its clinical significance.

There are still some limitations in the research. First, these participants recruited from outpatient department were relatively healthy with ambulatory activity. They were firstly assessed by the attending physician, then regular education was provided by the diabetes educator. Recruitments were conducted as the patients fit with the inclusion criteria and have no current acute illness of cerebrovascular accident, myocardial infarction, heart failure, renal failure, hepatic failure or psychiatric diseases. Therefore, the findings might not completely reflect the diabetic patients in this age (median 67.7 years). Second, this is an observational cross-sectional study of consecutive diabetic outpatients from a single hospital. Further prospective multicenter cohort study in large population will be required for assessing the clinical implication of the skinfold measurement, particular in evaluating the risks of cardiovascular disorders in diabetic patients. Finally, we only collected 59 participants in this study, which are relatively small for performing Bland-Altman analysis. According to the observation by González-Ruíz et al. [33], the upper limits of the differences between DXA and skinfold measurement are 18.3% in girls and 21.3% in boys. Therefore, provided we allowed the maximum difference to 22% in females and 23% in males and applied the mean and standard deviation of differences observed in our data (female: 8.74% and 4.45%; males: 7.22% and 4.92%) with selection of type I error as α = 0.05 and type II error as β = 0.2 (power: 80%), the minimum required sample size will be n = 27 in females and n = 19 in males via using the calculation presented by Lu et al. [42]. This paper serves as the first-stage research results and we will keep expand the sample sizes to decease the bias. Also, we will prospectively follow these diabetic patients to evaluate the subsequent trend of developing cardiometabolic disorders, such as myocardial infarction, ischemic stroke or cardiovascular death as the next-stage research.

In conclusion, our results indicate the 7-site skinfold measurement is not an interchangeable method for precisely measuring BF% due to its underestimation. Whereas, comparing to DXA as the reference method, it presents a strong correlation in assessing BF% and moderate to strong correlations in evaluating regional adiposity. The truncal and android skinfold thicknesses were positively correlated with multiple cardiometabolic risk factors. In contrary, the peripheral-to-android skinfold ratio and female thigh skinfold thickness are free of correlation with these risk factors. Therefore, the 7-site skinfold measurement might be a practical tool for estimating the cardiometabolic risks in Taiwanese diabetic outpatients.

## Supporting information

S1 Data (XLSX)
